# Gender and other factors associated with the use of hearing protection devices at work

**DOI:** 10.1590/S0034-8910.2015049005708

**Published:** 2015-10-05

**Authors:** Tatiane Costa Meira, Vilma Sousa Santana, Silvia Ferrite

**Affiliations:** I Programa de Pós-Graduação em Saúde Coletiva. Instituto de Saúde Coletiva. Universidade Federal da Bahia. Salvador, BA, Brasil; II Programa Integrado em Saúde Ambiental e do Trabalhador. Instituto de Saúde Coletiva. Universidade Federal da Bahia. Salvador, BA, Brasil; III Departamento de Fonoaudiologia. Instituto de Ciências da Saúde. Universidade Federal da Bahia. Salvador, BA, Brasil

**Keywords:** Ear Protective Devices, utilization, Hearing Loss, prevention & control, Occupational Risks, Gender and Health, Cross-Sectional Studies

## Abstract

**OBJECTIVE:**

To analyze whether sociodemographic, occupational, and health-related data are associated with the use of hearing protection devices at work, according to gender.

**METHODS:**

A cross-sectional study was conducted in 2006, using a random sample of 2,429 workers, aged between 18 and 65 years old, from residential sub-areas in Salvador, BA, Northeastern Brazil. Questionnaires were used to obtain sociodemographic, occupational, and health-related data. Workers who reported that they worked in places where they needed to shout in order to be heard were considered to be exposed to noise. Exposed workers were asked whether they used hearing protection devices, and if so, how frequently. Analyses were conducted according to gender, with estimates made about prevalence of the use of hearing protection devices, prevalence ratios, and their respective 95% confidence intervals.

**RESULTS:**

Twelve percent (12.3%) of study subjects reported that they were exposed to noise while working. Prevalence of the use of hearing protection devices was 59.3% for men and 21.4% for women. Men from higher socioeconomic levels (PR = 1.47; 95%CI 1.14;1.90) and who had previous audiometric tests (PR = 1.47; 95%CI 1.15;1.88) were more likely to use hearing protection devices. For women, greater perceived safety was associated with the use of protection devices (PR = 2.92; 95%CI 1.34;6.34). This perception was specifically related to the presence of supervisors committed to safety (PR = 2.09; 95%CI 1.04;4.21), the existence of clear rules to prevent workplace injuries (PR = 2.81; 95%CI 1.41;5.59), and whether they were informed about workplace safety (PR = 2.42; 95%CI 1.23;4.76).

**CONCLUSIONS:**

There is a gender bias regarding the use of hearing protection devices that is less favorable to women. The use of such devices among women is positively influenced by their perception of a safe workplace, suggesting that gender should be considered as a factor in hearing conservation programs.

## INTRODUCTION

Noise is one of the most common risk factors in work environments. It is the main modifiable cause of hearing loss in adults,^5^ and ranks third among the occupational hazards that generate the greatest number of years lived with disability.^a^ In 2010, in the European Union, 29.0% of workers were exposed to noise for at least a quarter of their daily working hours,^b^ while in the United States, between 1999 and 2004, 17.2% were exposed to noise over their entire work shift.^18^

Protecting workers from exposure to noise has been the target of international recommendations, such as ISO-1999^c^ and specific country regulations. Such recommendations consider factors ranging from the definition of acceptable noise limits to hearing conservation programs, which include the use of hearing protection devices (HPD).^d^ ^,^ ^e^

The World Health Organization (WHO) estimates that noise-induced hearing loss (NIHL) leading to irreversible cochlear damage accounts for 19.0% of all years lived with disability related to all work-related diseases and illnesses worldwide.^3^ NIHL can be prevented through collective measures, such as the modification or replacement of machines and equipment, and the use of acoustical barriers, silencers and enclosure, which are recognized as the best and most effective protection measures.^d^ When these are not technically feasible, while they are being installed, or used on an emergency basis, individual prevention measures are utilized to reduce the intensity of workers’ exposure to noise.^d^ ^,^ ^f^ These procedures reduce the risk of NIHL and other potentially adverse effects, such as insomnia, irritability, and increased heart rate and blood pressure.^12^ In Brazil, Regulatory Norm NR-6^f^ establishes the mandatory use of HPD when sound pressure levels exceed those defined by NR-15,^d^ i.e., 85 dB(A) for eight hours a day, or the equivalent.

Although HPD are mandatory, the prevalence of the use of these devices by workers exposed to noise is low: 42.2% of workers in Brazil^g^ and 65.7% in the United States.^18^ HPD use is positively associated with certain factors, such as increased noise levels in the workplace,^16^ ^,^ ^17^ being young,^17^ being influenced by peers and supervisors,^9^ ^,^ ^10^ and, particularly, being male, regardless of occupation.^17^ ^,^ ^18^

Women’s participation in the labor market has increased, especially in economic sectors and occupations traditionally considered to be predominantly male,^h^ ^,^ ^i^ which may have resulted in increased prevalence and/or intensity of exposure to noise. A study of women’s working and health conditions using data from the European Union^h^ showed that noise levels in women’s work environments are either neglected or poorly monitored, and prevention is not usually targeted by training and information. Such neglect arises from factors related to gender bias and because it occurs in non-industrial activities, such as services – particularly education, accommodation, food and beverage, among others.^h^ The WHO^j^ recommended that studies investigate noise exposure and its distinct effects on men and women, since this is rarely addressed in the literature. Studies on HPD use have not considered gender differences when identifying associated factors. The creation of proper hearing conservation programs cannot disregard information regarding the reasons for HPD use and should take gender differences into account.

This study aimed to analyze whether sociodemographic, occupational, and health-related data are associated with the use of hearing protection devices at work, according to gender.

## METHODS

This is a cross-sectional study based on data from a survey on working and health conditions, which used a sample of Salvador, BA, Northeastern Brazil, residents. The city has 2,675,656 residents (2010 census), making it the third most populated in the country. In 2010, it had a Human Development Index (HDI) of 0.759.^k^

The original study is a population-based prospective cohort study, which began in 2000, with visits repeated every two years until 2008. The data analyzed came from the fourth phase of the study, which was conducted in 2006, when data on hearing health and noise exposure were obtained. Cluster sampling was conducted in a single stage, with the selection of sub-areas, out of which each family was identified and recruited for the research. Trained field workers used individual questionnaires for interviews during home visits, in order to collect data related to sociodemography, lifestyle, working conditions, and health.

The study population comprises all subjects aged between 18 and 65 years old, who reported having a paid job and being exposed to noise in the workplace – 299 people in total. Noise exposure was defined by positive responses to two questions: “Have you ever worked in a very noisy environment, where you had to shout so that a colleague one meter away could hear you?”, which suggests noise exceeding 85 dB(A);^14^ and “Over the last 12 months, have you worked in an environment with that level of noise?”.

The outcome variable was the use of hearing protection devices when exposed to workplace noise (yes: regular or frequent use; no: seldom or never). The predictor variables were: 1) sociodemographic: age, ethnicity, marital status, education level, socioeconomic level (based on family assets: low - zero to two items; medium or high - three to nine items); occupational: occupational exposure to noise (in years), average hours of exposure per day, employment relationship (formal, when registered in the worker’s Employment Registration Book, or informal, when not), and perceived workplace safety; 3) hearing-related: self-reported hearing loss; tinnitus; previous audiometric tests; and 4) Self-reported health status, which was defined through a response to the question, “Between 0 and 10, what score would you give your health?” (score < 8 = poor, fair, or good; score ≥ 8 = great or excellent).

Regarding perceived workplace safety, responses to the following six questions were analyzed both separately and together: 1) “Are the health and safety of workers sufficiently protected in your workplace?”; 2) “Do your supervisors or bosses encourage you to protect yourself and prevent injuries?”; 3) “Do company owners spend money (invest) to make your workplace safe?”; 4) “Are clear rules established about what you should do to prevent workplace injuries?”; 5) “Is safety more important than production in the company where you work?”; 6) “Are you provided with information on workplace safety?” (adapted from the form used by Garcia et al,^7^ 2004). Answers “never”, “seldom”, and “sometimes” were taken as “no”, and “frequently” and “always”, as “yes”. The composite variable regarding perceived workplace safety corresponded to the total sum of scores, counting one point for each “yes” response and zero for each “no”, where each answer had the same weight. Categories for analysis were based on tertiles: 0 = poor; 1 to 4 = good; and 5 to 6 = very good.

Overall and specific prevalence of HPD use were estimated according to variable categories. Analyses were carried out separately by gender. The association measure was prevalence ratio (PR), to which 95% confidence intervals based on the Mantel-Haenszel method were used for statistical inference. Analyses were performed using SAS 9.2 statistical software.

The research was approved by the Ethics in Research Committee of the Hospital das Clínicas at the Universidade Federal da Bahia (Protocol 49, June 1^st^, 2000). All subjects signed informed consent forms.

## RESULTS

The study comprised 299 workers exposed to workplace noise out of the total cohort subjects (n = 2,429). Study subjects were more commonly young (76.6%), black (65.4%), in formal jobs (61.4%), male (60.9%) adults with at least high school education (67.6%). The majority had been exposed to occupational noise for at least five years (60.2%), eight hours a day or less (78.9%). Women exposed to occupational noise were less likely to be black (p = 0.0029), had more schooling (p = 0.0004), and were less likely to report previous audiometric tests (p < 0.0001). Men and women perceived workplace safety differently: 45.4% of the women considered their workplace safety to be “poor”, whereas only 20.5% men (p < 0.0001) expressed this opinion. There were no gender differences for the remaining variables.

Prevalence for use of HPD by workers exposed to noise was 44.5% (95%CI 38.9;50.1). Patterns of HPD use differed between genders; 59.3% of men and 21.4% of women reported HPD use (PR = 2.78; 95%CI 1.92;4.01). Regarding sociodemographic factors (Table 1), HPD use was associated with medium/high socioeconomic levels (compared to low) and with having had previous audiometric tests (compared to no tests) for men (Table 2). For women who perceived workplace safety as “very good” the prevalence of HPD use was three times greater than for those who reported working under “poor” safety conditions (Table 3). The influence of supervisors in preventing workplace injuries, the existence of clear rules to prevent them, and being informed about workplace safety were positively associated with HPD use in women.

**Table 1:** Prevalence (%) and prevalence ratio (PR) for the association between sociodemographic variables and use of hearing protection devices (HPD) in a population of workers exposed to workplace noise. Salvador, BA, Northeastern Brazil, 2006. (N = 299)

Variable	Female				Male			
	n	HPD use (%)	PR	95%CI	n	HPD use (%)	PR	95%CI
Total	117	21.4	1	13.9;28.8	182	59.3	1	52.2;66.5
Age (years)								
18 to 28	29	20.7	1.50	0.47;4.76	59	61.0	1.09	0.78;1.53
29 to 46	59	25.4	1.84	0.67;5.06	82	59.8	1.07	0.77;1.47
> 46	29	13.8	1	1	41	56.1	1	1
Ethnicity^a^								
Black/Mixed race	64	21.9	1	1	129	59.7	1	1
Non-black	52	21.1	0.97	0.48;1.95	50	56.0	0.94	0.71;1.25
Marital status								
Single	61	23.0	1	1	82	54.9	1	1
Married/Cohabiting	56	19.6	0.86	0.42;1.73	100	63.0	1.15	0.90;1.47
Education level								
Completed elementary school or less	24	12.5	1	1	73	58.9	1	1
Completed high school or above	93	23.7	1.89	0.62;5.80	109	59.6	1.01	0.79;1.29
Socioeconomic level^b^								
Low	49	26.5	1	1	85	48.2	1	1
Medium/High	63	17.5	0.66	0.32;1.34	90	71.1	1.47	1.14;1.90
Type of employment relationship^c^								
Informal	52	15.4	1	1	63	55.6	1	1
Formal	65	26.2	1.70	0.80;3.62	118	61.9	1.11	0.86;1.45

a No data for four subjects.

b No data for twelve subjects.

c No data for one subject.

**Table 2:** Prevalence (%) and prevalence ratio (PR) for the association between study variables and use of hearing protection devices (HPD) in a population of workers exposed to workplace noise. Salvador, BA, Northeastern Brazil, 2006. (N = 299)

Variable	Female				Male			
	n	HPD use (%)	PR	95%CI	n	HPD use (%)	PR	95%CI
Length of occupational exposure to noise								
< 5 years	48	27.1	1	1	71	59.2	1	1
≥ 5 years	69	17.4	0.64	0.32;1.28	111	59.5	1.00	0.79;1.29
Average hours of exposure per day								
≤ 8 hours/day	92	19.6	1	1	144	60.4	1	1
> 8 hours/day	25	28.0	1.43	0.67;3.04	38	55.3	0.91	0.67;1.25
Self-reported hearing loss								
No	78	21.8	1	1	137	61.3	1	1
Yes	39	20.5	0.94	0.45;1.99	45	53.3	0.87	0.64;1.18
Tinnitus								
No	103	21.4	1	1	168	58.9	1	1
Yes	14	21.4	1.00	0.34;2.92	14	64.3	1.09	0.72;1.64
Previous audiometric tests								
No	90	17.8	1	1	95	48.4	1	1
Yes	27	33.3	1.88	0.94;3.75	87	71.3	1.47	1.15;1.88
Self-reported health status (scale of 0 to 10)								
Poor/Fair/Good (0 to 7)	33	12.1	1	1	36	44.4	1	1
Great/Excellent (≥ 8)	84	25.0	2.06	0.77;5.55	146	63.0	1.42	0.96;2.09

**Table 3:** Prevalence (%) and prevalence ratio (PR) for the association between variables regarding perceived safety and use of hearing protection devices (HPD) in a population of workers exposed to workplace noise. Salvador, BA, Northeastern Brazil, 2006. (N = 299)

Variable	Female				Male			
	n	HPD use (%)	PR	95%CI	n	HPD use (%)	PR	95%CI
Perceived safety^a^ (composite variable)								
Poor (0)	49	16.3	1	1	33	54.5	1	1
Good (1 to 4)	38	15.8	0.97	0.37;2.55	59	62.7	1.15	0.80;1.66
Very Good (5 to 6)	21	47.6	2.92	1.34;6.34	69	63.8	1.17	0.82;1.67
p-trend				0.01				0.41
Are the health and safety of workers sufficiently protected at your workplace?^a,b^								
No	67	19.4	1	1	80	57.5	1	1
Yes	41	26.8	1.38	0.68;2.79	81	65.4	1.14	0.89;1.45
Do your supervisors or bosses encourage you to protect yourself and prevent injuries?^a,b^								
No	69	15.9	1	1	63	57.1	1	1
Yes	39	33.3	2.09	1.04;4.21	98	64.3	1.13	0.87;1.46
Do company owners spend money (invest) to make your workplace safe?^a,b^								
No	66	16.7	1	1	71	60.6	1	1
Yes	42	31.0	1.86	0.92;3.75	90	62.2	1.03	0.80;1.32
Are clear rules established about what you should do to prevent workplace injuries?^a,b^								
No	76	14.5	1	1	57	64.9	1	1
Yes	32	40.6	2.81	1.41;5.59	104	59.6	0.92	0.72;1.18
Is safety more important than production in the company where you work?^a,b^								
No	78	20.5	1	1	93	59.1	1	1
Yes	30	26.7	1.30	0.62;2.72	68	64.7	1.09	0.86;1.40
Are you provided with information on workplace safety?^a,b^								
No	80	16.2	1	1	69	56.5	1	1
Yes	28	39.3	2.42	1.23;4.76	92	65.2	1.15	0.89;1.49

a No data for thirty subjects.

b No = never, seldom, sometimes; Yes = frequently, always.

## DISCUSSION

Less than half of the workers exposed to noise used HPD; this was almost three times more common among men than women. The perceived degree of safety was associated with HPD use among women, with greater use related to greater perception of an unsafe work environment. Use of HPD was more likely to be reported by women who mentioned that they had committed supervisors, clearly stated safety rules, and access to safety information. Among men, medium or high socioeconomic levels and previous audiometric tests were associated factors.

Our estimated prevalence of HPD use is close to the finding of 41.4% from a study in the United States, based on data from 1981 to 1983, by the National Institute for Occupational Safety and Health (NIOSH).^4^ From 1999 to 2004, results from the National Health and Nutrition Examination Survey (NHANES) showed a prevalence of 65.7%.^18^ Despite evidence of a positive trend in the use of hearing protection in the United States, these results still indicate that one out of three workers exposed to noise did not use HPD.

Less HPD use by women – even when exposed to workplace noise – demonstrates that they are less protected, and more vulnerable, with increased risk of NIHL, compared with men. This gender bias merits attention. This finding confirms results from European studies, which found negligence in monitoring and prevention that demonstrated the invisibility of occupational noise exposure among women. Women have taken up jobs in industries that, although less obviously linked to NIHL risk factors, have been found to have high noise levels.^h^ This has been observed in countries with more advanced health regulations concerning labor-related health: in the United States, HPD use among women exposed to noise was 50.7%, compared with 68.9% among men.^18^ The more frequent use of hearing protection by men may be related to the job tasks and industries most frequently performed by men, in which high noise exposure levels are common, such as mining, construction, and manufacturing.^3^ Future analyses must consider specific occupations and industries, since these are important factors in noise exposure. This was not possible here, due to a small study sample composed of a general worker population.

In our study, factors associated with HPD use differed according to gender. Higher socioeconomic levels and having previous audiometric tests seem to favor the use of individual protection among men; among women, however, correlated factors were how their workplace was organized, namely their perception that it had a safety climate. Work in Brazil’s manufacturing industry is relatively well paid, and is known to have high levels of exposure to loud noises.^2^ This association may arise from a more hazardous work environment for NIHL, a more extensive adoption of protection measures, closer supervision of HPD use or compliance with legal regulations to protect workers’ health.

Previous audiometric testing seems to favor the use of HPD among men, which may also indicate better compliance to regulations, given presumably greater workplace noise. In Brazil, audiometric tests are mandatory for workers exposed to noise levels that exceed the legally permitted limits. Workers are also required to join hearing conservation programs.^l^ However, Lusk et al^11^ and Kim et al^10^ did not find that HPD use was associated with previous audiometric tests in the United States and South Korea, respectively.

The association between the positive aspects of perceived workplace safety and HPD use among women corroborates the findings of Edelson et al^6^ for workers of both genders. Women are more inclined towards sociability, which also applies to their workplaces, and are more influenced by recommendations to protect their health. Further, they are more likely to report health problems and more committed to healthy behavior, such as checkups, treatment compliance and other recommendations that promote their health.^13^ This may arise from behavioral and cultural patterns, as well as from awareness and commitment to their role as caregiver, particularly of children.^8^ It may also be a result of specific characteristics related to jobs and industries where women are usually engaged, which may have affected our findings. However, the small numbers in this study did not allow for further analysis.

The differences between men and women must be understood from a gender perspective. Women are affected by discrimination at work, earn lower wages than men (even when occupying similar jobs), face obstacles which limit their prospects of promotion, are less likely to be union members, and have less visibility and political capital in decision-making.^i^ Women are gradually achieving jobs in traditionally male areas, and have thus become increasingly involved in new production spaces.^i^ However, exposure to workplace hazards and preventive programs tend to be framed and analyzed from a male perspective. Hazards and their control are therefore frequently neglected or forgotten when they affect women.^h^ A European Union review of women’s working conditions^h^ demonstrated noise exposure in job posts not traditionally recognized as “hazardous” for men, such as education, health care and hospitality. It was also noted that women usually receive less training and fewer recommendations regarding HPD use than men.

Although age,^11^ ^,^ ^16^ ^,^ ^17^ education,^9^ and self-reported hearing loss^16^ have been found in association with HPD use, our results did not show this. This discrepancy may arise from methodological differences, particularly the heterogeneity of industries and jobs within the population studied.

As a tool for occupational noise control, the use of HPD is limited and not very effective, compared with collective interventions, which work independently of individual discretion or access to equipment for worker protection, and do not cause the discomfort or annoyance associated with anatomical or ergonomic adjustment difficulties. More appropriate recommendations are the control of noise emissions at their principal sources and of noise propagation in the workplace, and the implementation of actions to reduce worker exposure.^15^ However, such initiatives are not always feasible, causing the widespread use of HPD, given their low cost, reasonable effectiveness, and easy access.^10^

This research presents limitations related to statistical power, because of its small sample size. This is aggravated by a need to take into account gender, although it did enable the identification of distinct occupational factors between men and women. Given the few observations, it was not possible to include multinomial variables in the analysis, such as jobs or industries, nor to perform a more in-depth treatment of potential confounding variables or effect modifiers. Furthermore, our data comes from a study carried out with a different aim, thus reducing the scope of the available descriptor variables and other important causal factors, such as exposure intensity level. The HPD use did not take into account duration, or quality of adjustment, comfort, and level of protection. The advantages of this study include its relevance for prevention and its originality, because it did not merely describe the occurrence of clinical diagnoses in populations. Rather it focused on popularity, access, and compliance to a protection recommended against one of the most common labor-related illnesses, NIHL, which is responsible for a significant number of sensory disability cases worldwide. Since HPD use is mandatory for workers exposed to noise, the results of this study also demonstrate the extent of regulatory compliance. Organizational factors regarding risk management in work environments, such as the perceived safety investigated in this study, have not been sufficiently explored.

There is gender bias regarding protection against exposure to noise, namely in HPD use, which is less favorable to women than men. Gender, therefore, must be considered in hearing conservation programs. Although perceived safety and some of its components have only been associated with use of HPD among women, interventions based on these results may also contribute to the more frequent use of hearing protection devices by men, once such interventions are adjusted for male characteristics. Contributions can therefore be made to prevent the onset or worsening of NIHL, while better conditions for workers’ hearing health can be promoted.

## Footnotes

a World Health Organization. Global health risks: mortality and burden of disease attributable to selected major risks. Geneva; 2009 [cited 2014 Jun 18]. Available from: http://www.who.int/healthinfo/global_burden_disease/GlobalHealthRisks_report_ full.pdf

b The European Foundation for the Improvement of Living and Working Conditions. Fifth European Working Conditions Surveys. Luxembourg; 2012 [cited 2015 Apr 30]. Available from: http://www.eurofound.europa.eu/sites/default/files/ef_files/pubdocs/2011/82/en/1/EF1182EN.pdf

c International Organization for Standardization. Determination of occupational noise exposure and estimation of noise-induced hearing impairment - ISO 1990:1999. 2.ed. Geneva; 1990 [cited 2015 Apr 30]. Available from: http://www.iso.org/iso/catalogue_detail.htm?csnumber=6759

d Ministério do Trabalho e Emprego. Norma Regulamentadora nº 15: atividades e operações insalubres. Brasília (DF); 1978 [cited 2015 Apr 28]. Available from: http://portal.mte.gov.br/data/files/8A7C816A47594D040147D14EAE840951/NR-15%20(atualizada%202014).pdf

e National Institute for Occupational Safety and Health. Criteria for a Recommended Standard: occupational noise exposure: revised criteria 1998. Cincinnati: NIOSH; CDC; 1998 [cited 2014 Jun 18]. Available from: http://www.cdc.gov/niosh/docs/98-126/pdfs/98-126.pdf

f Ministério do Trabalho e Emprego. Norma Regulamentadora Nº 6: Equipamento de Proteção Individual – EPI. Brasília (DF); 1978 [cited 2015 Apr 28]. Available from: http://portal.mte.gov.br/data/files/FF8080814CD7273D014D34C6B18C79C6/NR-06%20(atualizada)%202015.pdf

g Ferrite S. Epidemiologia da perda auditiva em adultos trabalhadores [thesis]. Salvador (BA): Instituto de Saúde Coletiva da Universidade Federal da Bahia; 2009 [cited 2015 Apr 28]. Available from: https://repositorio.ufba.br/ri/handle/ri/10892

h European Agency for Safety and Health at Work. New risks and trends in the safety and health of women at work. Luxembourg: Publications Office for the European Union; 2013. DOI:10.2802/69206

i Messing K, Östlin P. Gender equality, work and health: a review of the evidence. Geneva: World Health organization; 2006 [cited 2014 Jun 18] Available from: http://www.who.int/gender/documents/Genderworkhealth.pdf?ua=

j Concha-Barrientos M, Campbell-Lendrum D, Steenland NK. Occupational noise: assessing the burden of disease from work-related hearing impairment at national and local levels. Geneva: World Health Organization; 2004 [cited 2015 Apr 28]. (WHO Environmental Burden of Disease Series, 9). Available from: http://www.who.int/quantifying_ehimpacts/publications/en/ebd9.pdf

k Instituto Brasileiro de Geografia e Estatística. Cidades. Brasília (DF); 2014 [cited 2014 Jun 18]. Available from: http://www.cidades.ibge.gov.br/xtras/home.php

l Ministério do Trabalho e Emprego, Secretaria de Saúde e Segurança no Trabalho. Portaria nº 19 de 9 de abril de 1998. Diretrizes e parâmetros mínimos para avaliação a acompanhamento da audição em trabalhadores expostos a níveis de pressão sonora elevados. *Diario Oficial Uniao*. 22 abr 1998; Seção 1:64-6.
