# Supplementary material for: Gender and other factors associated with the use of hearing protection devices at work
Source: Rev Saude Publica. 2015 Oct 5;49:76. doi: 10.1590/S0034-8910.2015049005708 (PMC4603261; doi:10.1590/S0034-8910.2015049005708)
Supplement: Translation

# Translation: Gênero e fatores associados ao uso de equipamento de proteção auditiva no trabalho

**Authors:** Tatiane Costa Meira, Vilma Sousa Santana, Silvia Ferrite

**Affiliations:** I Programa de Pós-Graduação em Saúde Coletiva. Instituto de Saúde Coletiva. Universidade Federal da Bahia. Salvador, BA, Brasil; II Programa Integrado em Saúde Ambiental e do Trabalhador. Instituto de Saúde Coletiva. Universidade Federal da Bahia. Salvador, BA, Brasil; III Departamento de Fonoaudiologia. Instituto de Ciências da Saúde. Universidade Federal da Bahia. Salvador, BA, Brasil

**Keywords:** Dispositivos de Proteção Auditiva, utilização, Perda Auditiva, prevenção & controle, Riscos Ocupacionais, Gênero e Saúde, Estudos Transversais

## Abstract

**OBJETIVO:**

Analisar se dados sociodemográficos, ocupacionais e de saúde estão associados ao uso de equipamento de proteção auditiva no trabalho, segundo gênero.

**MÉTODOS:**

Estudo transversal com amostra aleatória de 2.429 trabalhadores de 18 a 65 anos de subáreas de base domiciliar em Salvador, BA, no ano de 2006. Foram aplicados questionários para obtenção de dados sociodemográficos, ocupacionais e de saúde. Foram considerados expostos ao ruído aqueles que relataram trabalhar em local onde era necessário gritar para ser ouvido. Os trabalhadores expostos foram questionados sobre uso e regularidade do uso de equipamento de proteção auditiva. A análise foi conduzida por gênero, estimando-se a prevalência do uso do equipamento de proteção auditiva, razões de prevalência e os respectivos intervalos de 95% de confiança.

**RESULTADOS:**

Entre os participantes do estudo, 12,3% referiram trabalhar expostos ao ruído. A prevalência do uso do equipamento de proteção auditiva foi 59,3% e 21,4%, para homens e mulheres, respectivamente. Entre os homens, maior nível socioeconômico (RP = 1,47; IC95% 1,14;1,90) e ter realizado audiometria (RP = 1,47; IC95% 1,15;1,88) foram fatores associados ao uso do equipamento. Entre as mulheres, a percepção de maior segurança associou-se ao uso do equipamento (RP = 2,92; IC95% 1,34;6,34). Essa percepção deveu-se, especialmente, à presença de supervisores comprometidos com a segurança (RP = 2,09; IC95% 1,04;4,21), à existência de regras claras para evitar acidentes de trabalho (RP = 2,81; IC95% 1,41;5,59) e ao recebimento de informações sobre segurança no trabalho (RP = 2,42; IC95% 1,23;4,76).

**CONCLUSÕES:**

Há um viés de gênero em relação ao uso do equipamento de proteção auditiva menos favorável às mulheres em comparação com os homens. O uso do equipamento entre mulheres é influenciado positivamente pela percepção de um ambiente de trabalho seguro, sugerindo que o gênero precisa ser considerado nos programas de conservação auditiva.

## INTRODUÇÃO

O ruído é um dos fatores de risco mais comuns nos ambientes de trabalho. É a principal causa modificável para a perda auditiva em adultos^5^ e ocupa o terceiro lugar no ranking dos fatores ocupacionais que mais geram anos vividos com incapacidade.^a^ Em 2010, 29,0% dos trabalhadores estavam expostos ao ruído em pelo menos 1/4 das horas diárias de trabalho na União Europeia,^b^ e 17,2% estavam expostos durante todo o turno de trabalho, nos Estados Unidos, entre 1999 e 2004.^18^

A proteção dos trabalhadores contra a exposição ao ruído é objeto de recomendações internacionais, a exemplo da ISO-1999^c^ e de regulamentações específicas de cada país. Essas recomendações compreendem desde a definição de limites de tolerância até a implantação de programas de conservação auditiva, que incluem o uso do equipamento de proteção auditiva (EPA).^d^ ^,^ ^e^

A Organização Mundial da Saúde (OMS) estima que a perda auditiva induzida pelo ruído (PAIR), que resulta de uma lesão coclear irreversível, é responsável por 19,0% dos anos vividos com incapacidade por todas as doenças e agravos relacionados ao trabalho no mundo.^3^ A PAIR é passível de prevenção com as medidas coletivas, como a modificação ou substituição de máquinas e equipamentos, uso de barreiras, silenciadores e enclausuramento de máquinas, entre outras, reconhecidas como as melhores e mais efetivas medidas de proteção.^d^ Quando essas medidas forem tecnicamente inviáveis, estiverem sendo implementadas ou em caráter emergencial, medidas de prevenção individuais são adotadas para auxiliar na redução do nível de ruído que atinge o trabalhador.^d^ ^,^ ^f^ Isso reduz o risco do desencadeamento da PAIR e de outros efeitos adversos potenciais como a insônia, a irritabilidade e o aumento da frequência cardíaca e da pressão arterial.^12^ No Brasil, a NR-6^f^ estabelece que o uso do EPA é obrigatório quando os níveis de pressão sonora são superiores aos definidos pela NR-15,^d^ i.e., 85 dB(A) por oito horas diárias ou dose equivalente.

Apesar da obrigatoriedade do uso do EPA, a prevalência do uso desse equipamento entre trabalhadores expostos ao ruído é baixa: 42,2% entre trabalhadores no Brasil^g^ e 65,7% nos Estados Unidos.^18^ O uso do EPA associa-se positivamente a alguns fatores, como maior nível de ruído no ambiente laboral,^16^ ^,^ ^17^ ser jovem,^17^ ter influência de pares e supervisores,^9^ ^,^ ^10^ e, em especial, ser do sexo masculino, independentemente da ocupação.^17^ ^,^ ^18^

A participação feminina no mercado de trabalho aumenta, em especial em setores de atividade econômica e ocupações tradicionalmente consideradas masculinas,^h^ ^,^ ^i^ o que pode traduzir-se em maiores níveis de prevalência e/ou de intensidade de exposição ao ruído. Estudo sobre condição de trabalho e saúde da mulher, com dados da União Europeia,^h^ mostrou que o ruído em ambientes de trabalho entre as mulheres é negligenciado, pouco monitorado e a sua prevenção não costuma ser alvo de treinamento e informação. Essa negligência decorre de aspectos relacionados à discriminação de gênero e por ocorrer em atividades não industriais, como a de serviços, especialmente educação, restaurantes, bares e hotelaria, dentre outros.^h^ A OMS^j^ recomendou que os estudos investiguem a exposição ao ruído e seus efeitos separadamente entre homens e mulheres, o que vem sendo pouco observado na literatura. Estudos sobre o uso de EPA não consideraram as diferenças de gênero na identificação de fatores associados. A elaboração de programas de conservação auditiva adequados não pode prescindir de informações sobre as causas do uso de EPA, levando em consideração diferenças de gênero.

O objetivo deste estudo foi analisar se dados sociodemográficos, ocupacionais e de saúde estão associados ao uso de equipamento de proteção auditiva no trabalho, segundo gênero.

## MÉTODOS

Estudo transversal com base nos dados de pesquisa sobre condições de trabalho e saúde realizada com uma amostra da população residente em Salvador, BA. Com 2.675.656 habitantes (censo de 2010), é a terceira cidade mais populosa do País e apresentava Índice de Desenvolvimento Humano (IDH) de 0,759, em 2010.^k^

O estudo original é uma coorte prospectiva de base populacional, iniciada em 2000, com revisitas a cada dois anos, até 2008. Foram analisados dados da quarta fase do estudo, realizada em 2006, quando foram obtidos dados sobre saúde auditiva e exposição ao ruído. A amostragem da coorte foi do tipo aleatória por conglomerados, em estágio único, com seleção de subáreas, das quais as famílias identificadas eram recrutadas para a pesquisa. Entrevistadores treinados aplicaram questionários individuais em visitas domiciliares para coleta de dados sociodemográficos, hábitos de vida, condições de trabalho e saúde.

A população do presente estudo compreendeu os indivíduos entre 18 e 65 anos de idade que referiram trabalho remunerado e exposição a ruído no seu ambiente de trabalho, totalizando 299 pessoas. A exposição ao ruído foi definida a partir da resposta positiva a duas perguntas: “Você já trabalhou em algum ambiente com muito barulho onde seria preciso gritar para que um colega a um metro de distância pudesse ouvir?”, que sugere ruído em intensidade que excede 85 dB(A)^14^ e “Nos últimos 12 meses, você trabalhou em algum ambiente com esse tipo de barulho?”.

A variável dependente foi o uso do equipamento de proteção auditiva quando exposto ao ruído no trabalho (sim: uso regular ou frequente; não: raramente ou nunca). As variáveis independentes foram: 1) sociodemográficas: idade, cor da pele, situação conjugal, nível de escolaridade, nível socioeconômico (com base no número de posses da família: baixa - zero a dois itens; média ou alta - três a nove itens); 2) ocupacionais: exposição ocupacional ao ruído (em anos), número médio de horas diárias exposto, tipo de contrato de trabalho (formal, quando com registro em carteira de trabalho, ou informal, na ausência deste) e clima de segurança no local de trabalho; 3) auditivas: perda auditiva autorreferida, sensação de zumbido, ter alguma vez realizado audiometria; e 4) condição de saúde autopercebida, definida a partir da pergunta: “Que nota, de zero a 10, você daria à sua saúde?” (nota < 8 = ruim, regular ou boa; nota ≥ 8 = ótima ou excelente).

Para percepção da segurança no ambiente de trabalho, respostas correspondentes a seis perguntas foram analisadas individualmente e de modo combinado: 1) No meu trabalho, a saúde e a segurança dos trabalhadores estão suficientemente protegidas?; 2) Os supervisores ou chefes encorajam a gente a se proteger e evitar acidentes?; 3) Os donos da empresa gastam dinheiro (investem) para que o ambiente de trabalho seja seguro?; 4) Existem regras bem claras sobre o que devemos fazer para evitar acidentes de trabalho?; 5) Na empresa em que trabalho é mais importante a segurança do que a produção?; 6) Eu recebo informações sobre segurança no trabalho? (adaptadas do instrumento utilizado por Garcia et al,^7^ 2004). As categorias, “nunca”, “raramente” e “algumas vezes” foram consideradas como “não”, e “frequentemente” e “sempre”, como “sim”. A variável composta referente à percepção de segurança no ambiente de trabalho correspondeu ao somatório das respostas, considerando um ponto para cada “sim” e zero para cada “não”, com peso equivalente para todas as perguntas. Foram construídas categorias de análise segundo os tercis da distribuição: 0 = ruim; 1 a 4 = boa; e 5 a 6 = muito boa.

Foram estimadas as prevalências do uso do EPA, geral e específica, de acordo com as categorias das variáveis. As análises foram realizadas separadamente por gênero. A medida de associação utilizada foi razão de prevalência (RP) com intervalos de 95% de confiança, calculados pelo método de Mantel-Haenszel.

As análises foram conduzidas utilizando-se o programa estatístico SAS 9.2.

O estudo foi aprovado pelo Comitê de Ética do Hospital das Clínicas da Universidade Federal da Bahia (Protocolo 49, de 1/6/2000). Todos os participantes assinaram termo de consentimento livre e esclarecido.

## RESULTADOS

O estudo compreendeu 299 trabalhadores expostos ao ruído no trabalho, identificados entre o total de sujeitos da coorte (n = 2.429). Predominaram indivíduos do gênero masculino (60,9%), adultos jovens (76,6%), de pele negra (65,4%), com, pelo menos, o segundo grau completo (67,6%) e contrato formal de trabalho (61,4%). A maioria estava exposta a ruído ocupacional por, pelo menos, cinco anos (60,2%), por oito horas diárias ou menos (78,9%). Mulheres expostas ao ruído eram menos comumente de cor negra (p = 0,0029), tinham maior escolaridade (p = 0,0004) e referiram menos frequentemente ter realizado audiometria (p < 0,0001). O clima de segurança no ambiente de trabalho foi percebido diferentemente entre homens e mulheres: 45,4% das mulheres consideraram o clima de segurança “ruim”, em contraste com 20,5% dos homens (p < 0,0001). Para as demais variáveis, não houve diferenças entre os gêneros.

A prevalência do uso do EPA entre os trabalhadores expostos ao ruído foi de 44,5% (IC95% 38,9;50,1). Houve diferença no uso do EPA entre os gêneros, referido por 59,3% dos homens e por 21,4% das mulheres (RP = 2,78; IC95% 1,92;4,01). Entre os fatores sociodemográficos (Tabela 1), houve associação com o uso do EPA para o nível socioeconômico médio/alto, comparando-se aqueles com nível socioeconômico baixo, no gênero masculino; e ter realizado audiometria entre os homens, comparando-se aos que nunca realizaram (Tabela 2). Entre as mulheres, no grupo clima de segurança “muito bom”, a prevalência do uso do EPA foi três vezes maior do que no grupo com clima de segurança “ruim” (Tabela 3). Influência do supervisor para evitar acidentes de trabalho, existência de regras claras para evitar acidentes e receber informações sobre segurança no trabalho apresentaram associação positiva com o uso do EPA entre as mulheres.

**Tabela 1:** Prevalência (%) e razão de prevalência (RP) para a associação entre variáveis sociodemográficas e o uso de equipamento de proteção auditiva (EPA) em população de trabalhadores expostos ao ruído no trabalho. Salvador, BA, 2006. (N = 299)

Variável	Mulher				Homem			
	n	Uso do EPA (%)	RP	IC95%	n	Uso do EPA (%)	RP	IC95%
Total	117	21,4(%)	1	13,9;28,8	182	59,3	1	52,2;66,5
Idade (anos)								
18 a 28	29	20,7	1,50	0,47;4,76	59	61,0	1,09	0,78;1,53
29 a 46	59	25,4	1,84	0,67;5,06	82	59,8	1,07	0,77;1,47
> 46	29	13,8	1	1	41	56,1	1	1
Cor da pele^a^								
Negro/Mulato	64	21,9	1	1	129	59,7	1	1
Não negro	52	21,1	0,97	0,48;1,95	50	56,0	0,94	0,71;1,25
Situação conjugal								
Solteiro	61	23,0	1	1	82	54,9	1	1
Casado/Consensual	56	19,6	0,86	0,42;1,73	100	63,0	1,15	0,90;1,47
Nível de escolaridade								
1º grau completo ou menor	24	12,5	1	1	73	58,9	1	1
2º grau completo ou maior	93	23,7	1,89	0,62;5,80	109	59,6	1,01	0,79;1,29
Nível socioeconômico^b^								
Baixo	49	26,5	1	1	85	48,2	1	1
Médio/Alto	63	17,5	0,66	0,32;1,34	90	71,1	1,47	1,14;1,90
Tipo de contrato de trabalho^c^								
Informal	52	15,4	1	1	63	55,6	1	1
Formal	65	26,2	1,70	0,80;3,62	118	61,9	1,11	0,86;1,45

a Dados ausentes para quatro indivíduos.

b Dados ausentes para doze indivíduos.

c Dados ausentes para um indivíduo.

**Tabela 2:** Prevalência (%) e razão de prevalência (RP) para a associação entre variáveis do estudo e o uso do equipamento de proteção auditiva (EPA) em uma população de trabalhadores expostos ao ruído no trabalho. Salvador, BA, 2006. (N = 299)

Variável	Mulher				Homem			
	n	Uso do EPA (%)	RP	IC95%	n	Uso do EPA (%)	RP	IC95%
Duração da exposição ocupacional ao ruído								
< 5 anos	48	27,1	1	1	71	59,2	1	1
≥ 5 anos	69	17,4	0,64	0,32;1,28	111	59,5	1,00	0,79;1,29
Número médio de horas diárias exposto								
≤ 8 horas/dia	92	19,6	1	1	144	60,4	1	1
> 8 horas/dia	25	28,0	1,43	0,67;3,04	38	55,3	0,91	0,67;1,25
Perda auditiva autorreferida								
Não	78	21,8	1	1	137	61,3	1	1
Sim	39	20,5	0,94	0,45;1,99	45	53,3	0,87	0,64;1,18
Sensação de zumbido								
Não	103	21,4	1	1	168	58,9	1	1
Sim	14	21,4	1,00	0,34;2,92	14	64,3	1,09	0,72;1,64
Ter realizado audiometria								
Não	90	17,8	1	1	95	48,4	1	1
Sim	27	33,3	1,88	0,94;3,75	87	71,3	1,47	1,15;1,88
Condição de saúde autopercebida (escala de 0 a 10)								
Ruim/Regular/Boa (0 a 7)	33	12,1	1	1	36	44,4	1	1
Ótima/Excelente (≥ 8)	84	25,0	2,06	0,77;5,55	146	63,0	1,42	0,96;2,09

**Tabela 3:** Prevalência (%) e razão de prevalência (RP) para a associação entre as variáveis do clima de segurança e o uso do equipamento de proteção auditiva (EPA) em uma população de trabalhadores expostos ao ruído no trabalho. Salvador, BA, 2006. (N = 299)

Variável	Mulher				Homem			
	n	Uso do EPA (%)	RP	IC95%	n	Uso do EPA (%)	RP	IC95%
Clima de segurança^a ^(variável composta)								
Ruim (0)	49	16,3	1	1	33	54,5	1	1
Bom (1 a 4)	38	15,8	0,97	0,37;2,55	59	62,7	1,15	0,80;1,66
Muito bom (5 a 6)	21	47,6	2,92	1,34;6,34	69	63,8	1,17	0,82;1,67
*p*-tendência				0,01				0,41
No meu trabalho, a saúde e a segurança dos trabalhadores estão suficientemente protegidas^a,b^								
Não	67	19,4	1	1	80	57,5	1	1
Sim	41	26,8	1,38	0,68;2,79	81	65,4	1,14	0,89;1,45
Os supervisores ou chefes encorajam a gente a se proteger e evitar acidentes^a,b^								
Não	69	15,9	1	1	63	57,1	1	1
Sim	39	33,3	2,09	1,04;4,21	98	64,3	1,13	0,87;1,46
Os donos da empresa gastam dinheiro (investem) para que o ambiente de trabalho seja seguro^a,b^								
Não	66	16,7	1	1	71	60,6	1	1
Sim	42	31,0	1,86	0,92;3,75	90	62,2	1,03	0,80;1,32
Existem regras bem claras sobre o que devemos fazer para evitar acidentes de trabalho^a,b^								
Não	76	14,5	1	1	57	64,9	1	1
Sim	32	40,6	2,81	1,41;5,59	104	59,6	0,92	0,72;1,18
Na empresa em que trabalho é mais importante a segurança do que a produção?^a,b^								
Não	78	20,5	1	1	93	59,1	1	1
Sim	30	26,7	1,30	0,62;2,72	68	64,7	1,09	0,86;1,40
Eu recebo informações sobre segurança no trabalho^a,b^								
Não	80	16,2	1	1	69	56,5	1	1
Sim	28	39,3	2,42	1,23;4,76	92	65,2	1,15	0,89;1,49

a Dados ausentes para 30 indivíduos.

b Não = nunca, raramente, algumas vezes; Sim = frequentemente, sempre.

## DISCUSSÃO

Menos da metade dos trabalhadores expostos ao ruído utilizava o EPA, comportamento quase três vezes mais comum entre homens do que entre mulheres. O grau do clima de segurança associou-se ao uso do EPA entre mulheres, com gradiente dose-resposta. O uso do EPA foi mais comum entre aquelas que referiram supervisores comprometidos, regras claras e recebimento de informações sobre segurança. Entre os homens, foram fatores associados o nível socioeconômico médio ou alto e ter realizado audiometria.

A prevalência do uso do EPA neste estudo foi comparável com a dos Estados Unidos, 41,4%, calculada com dados das pesquisas do *National Institute for Occupational Safety and Health* (NIOSH) de 1981 a 1983.^4^ Essa mesma medida alcançou 65,7%, de 1999 a 2004, utilizando-se dados do *National Health and Nutrition Examination Survey* (NHANES).^18^ Embora evidente a evolução positiva do uso da proteção auditiva nos Estados Unidos, os resultados indicavam que um em cada três trabalhadores expostos ao ruído não usava o EPA.

O menor uso de EPA pelas mulheres em comparação aos homens, mesmo quando expostas ao ruído no trabalho, mostra sua condição de menor proteção, maior vulnerabilidade e risco potencial de PAIR. Trata-se, portanto, de um viés de gênero que merece atenção. Esse achado confirma as interpretações dos resultados de estudos europeus que mencionaram a negligência do monitoramento e da prevenção, e a invisibilidade da exposição ao ruído entre mulheres. Estas assumem ocupações e atividades econômicas menos óbvias para esse fator de risco da PAIR, mas para as quais são observados níveis elevados de ruído.^h^ Isso se repete mesmo em países avançados quanto à saúde do trabalhador: nos Estados Unidos, o uso de EPA entre as mulheres expostas ao ruído foi de 50,7%, em contraste com 68,9% dos homens.^18^ O maior uso da proteção auditiva entre os homens pode relacionar-se às atividades econômicas e ocupações em que predominam o gênero masculino, nas quais prevalecem níveis elevados de exposição ao ruído, como a mineração, a construção e indústria manufatureira.^3^ Análises futuras devem considerar o ramo de atividade e a ocupação, importantes determinantes da exposição ao ruído. Isso não foi possível neste estudo devido aos pequenos números, por se tratar de uma população geral de trabalhadores.

No presente estudo, os fatores associados ao uso do EPA foram diferentes de acordo com o gênero. Maior nível socioeconômico e ter realizado audiometria parecem favorecer o uso de proteção individual entre os homens, enquanto esses fatores relacionaram-se à organização do trabalho, especificamente, à percepção de ambiente de trabalho seguro, entre as mulheres. O trabalho na indústria manufatureira no Brasil é relativamente bem remunerado, e são conhecidos os elevados índices de exposição e da intensidade do ruído nessa indústria.^2^ Assim, essa associação pode resultar da atividade econômica mais perigosa para a PAIR, ou da maior extensão da adoção de medidas de proteção, maior supervisão do uso do EPA e cumprimento de normas legais estabelecidas para a proteção da saúde dos trabalhadores.

A realização prévia de audiometria parece favorecer o uso do EPA entre os homens, o que pode indicar também maior cumprimento de normas, além de ruído mais elevado nos locais de trabalho. No Brasil, a avaliação da audição por meio da audiometria é obrigatória para os trabalhadores com exposição acima do limite de tolerância estabelecido pela Legislação, assim como a inclusão em Programas de Conservação Auditiva.^l^ Já Lusk et al^11^ e Kim et al^10^ não encontraram associação de antecedentes de audiometria com o uso do EPA nos Estados Unidos e na Coréia do Sul, respectivamente.

A associação entre aspectos positivos do clima de segurança no ambiente de trabalho e o uso do EPA em mulheres confirmam os achados de Edelson et al^6^ entre trabalhadores de ambos os gêneros. Mulheres são mais conectadas à sociabilidade, o que se aplica também ao ambiente de trabalho, e se deixam influenciar por recomendações relativas à proteção da sua saúde. Também relatam mais comumente problemas de saúde e se engajam em comportamentos de saúde como o comparecimento a visitas médicas de rotina, realização de *checkups *e aderência a tratamentos e outras recomendações relativas à promoção da saúde.^13^ Isso pode resultar de padrões comportamentais e culturais, bem como da consciência e comprometimento com o seu papel de cuidadora, em especial dos filhos.^8^ Pode também ter sido determinado por peculiaridades dos ramos de atividade e das ocupações em que trabalhavam as mulheres, influenciando nos resultados, mas os pequenos números não permitiram análise mais aprofundada.

Essas diferenças entre homens e mulheres devem ser compreendidas a partir das questões de gênero. Mulheres são alvo de discriminação no trabalho, recebem menores salários que os homens, mesmo quando se encontram em postos semelhantes de trabalho, são afetadas por barreiras que limitam sua progressão para cargos hierarquicamente superiores, participam em menor proporção em sindicatos, e têm menor visibilidade e capital político nas decisões.^i^ Gradativamente, mulheres ocupam espaços em atividades nas quais se concentravam homens e ampliam a diversidade de ocupações e ramos de atividade econômica em que se inserem.^i^ Todavia, exposições em local de trabalho e ações preventivas tendem a ser vistas e analisadas na perspectiva masculina, e negligenciadas ou despercebidas quando se tratam de mulheres.^h^ Estudo de revisão da União Europeia sobre condições de trabalho entre mulheres^h^ mostra a exposição ao ruído em ramos que usualmente não são considerados como “de risco” entre os homens, a exemplo das áreas de educação, saúde, hotelaria e restaurantes. Destacam ainda que mulheres recebem menos treinamentos ou recomendações para uso do EPA comparando-se aos homens.

Embora a idade,^11^ ^,^ ^16^ ^,^ ^17^ nível de escolaridade^9^ e perda auditiva autorreferida^16^ sejam apontados como associados ao uso do EPA, tais associações não foram encontradas neste estudo. Essa inconsistência pode decorrer de diferenças nas abordagens metodológicas, especialmente na composição e distribuição dos ramos de atividade e ocupações subjacentes nas populações de estudo.

O uso do EPA é uma medida de controle do ruído ocupacional limitada e de reduzida efetividade, em comparação às de caráter coletivo que independem da decisão dos sujeitos, do acesso ao equipamento e não se encontram relacionadas a desconforto ou incômodos associados a problemas de ajustes anatômicos e ergonômicos. Recomendam-se, mais apropriadamente, o controle da emissão do ruído na fonte principal de exposição, da sua propagação no ambiente de trabalho e ações visando a redução do tempo de exposição do trabalhador.^15^ Todavia, essas medidas nem sempre são factíveis. Assim, o EPA tem sido amplamente utilizado, também por seu menor custo, relativa efetividade e fácil acesso.^10^

Esta pesquisa possui limitações relacionadas ao poder estatístico em função do pequeno número amostral. Isso é agravado pela necessidade da análise estratificada por gênero, mas que permitiu a identificação de fatores que se mostraram distintos entre homens e mulheres. Com poucas observações, a análise não pôde ser expandida para o exame de variáveis multinomiais, como o ramo de atividade econômica e a ocupação, e aprofundamento na avaliação de relações mais complexas, com o ajuste para variáveis de confusão e a consideração de modificadores de efeito potenciais. Além disso, o estudo foi conduzido com dados de uma pesquisa com foco em outro objeto, o que reduz o escopo de variáveis descritoras disponíveis e outros importantes determinantes como o nível de intensidade da exposição. A variável relativa ao uso do EPA foi medida de modo simples, não considerando o tempo de uso, qualidade do ajustamento, conforto e proteção. Dentre as vantagens deste estudo, estão a sua relevância e originalidade, pois avança ao ir além de diagnósticos de agravos à saúde. Focalizou a popularidade, o acesso e a adesão ao uso de uma medida de proteção para uma das mais comuns enfermidades relacionadas ao trabalho, a PAIR, responsável por expressivo número de casos de incapacidade sensorial em todo o mundo. Como o EPA é de uso compulsório entre expostos ao ruído, os resultados deste estudo também mostram a extensão com que normas reguladoras são cumpridas. Fatores organizacionais relativos à gestão do risco em ambientes de trabalho têm sido pouco explorados, como o clima de segurança, investigado neste estudo.

Há um viés de gênero em relação à proteção da exposição ao ruído, especificamente, para o uso do EPA, menos favorável às mulheres em comparação com os homens. O gênero, portanto, precisa ser considerado nos Programas de Conservação Auditiva. Embora o grau do clima de segurança e alguns dos seus componentes tenham se associado ao uso do EPA, apenas entre mulheres, é possível que intervenções pautadas nesse resultado, com ajustes para as especificidades masculinas, possam também contribuir para melhor adesão à proteção auditiva entre os homens. Assim, pode-se contribuir para a prevenção do desencadeamento ou do agravamento da PAIR e promover melhores condições de saúde auditiva para os trabalhadores.

## Footnotes

a World Health Organization. Global health risks: mortality and burden of disease attributable to selected major risks. Geneva; 2009 [citado 2014 jun 18]. Disponível em: http://www.who.int/healthinfo/global_burden_disease/GlobalHealthRisks_report_ full.pdf

b The European Foundation for the Improvement of Living and Working Conditions. Fifth European Working Conditions Surveys. Luxembourg; 2012 [citado 2015 abr 30]. Disponível em: http://www.eurofound.europa.eu/sites/default/files/ef_files/pubdocs/2011/82/en/1/EF1182EN.pdf

c International Organization for Standardization. Determination of occupational noise exposure and estimation of noise-induced hearing impairment - ISO 1990:1999. 2.ed. Geneva; 1990 [citado 2015 abr 30]. Disponível em: http://www.iso.org/iso/catalogue_detail.htm?csnumber=6759

d Ministério do Trabalho e Emprego. Norma Regulamentadora Nº 15: atividades e operações insalubres. Brasília (DF); 1978 [citado 2015 abr 28]. Disponível em: http://portal.mte.gov.br/data/files/8A7C816A47594D040147D14EAE840951/NR-15%20(atualizada%202014).pdf

e National Institute for Occupational Safety and Health. Criteria for a Recommended Standard: occupational noise exposure: revised criteria 1998. Cincinnati: NIOSH; CDC; 1998 [citado 2014 jun 18]. Disponível em: http://www.cdc.gov/niosh/docs/98-126/pdfs/98-126.pdf

f Ministério do Trabalho e Emprego. Norma Regulamentadora Nº 6: Equipamento de Proteção Individual – EPI. Brasília (DF); 1978 [citado 2015 abr 28]. Disponível em: http://portal.mte.gov.br/data/files/FF8080814CD7273D014D34C6B18C79C6/NR-06%20(atualizada)%202015.pdf

g Ferrite S. Epidemiologia da perda auditiva em adultos trabalhadores [tese de doutorado]. Salvador (BA): Instituto de Saúde Coletiva da Universidade Federal da Bahia; 2009 [citado 2015 abr 28]. Disponível em: https://repositorio.ufba.br/ri/handle/ri/10892

h European Agency for Safety and Health at Work. New risks and trends in the safety and health of women at work. Luxembourg: Publications Office for the European Union; 2013. DOI:10.2802/69206

i Messing K, Östlin P. Gender equality, work and health: a review of the evidence. Geneva: World Health organization; 2006 [citado 2014 jun 18] Disponível em: http://www.who.int/gender/documents/Genderworkhealth.pdf?ua=

j Concha-Barrientos M, Campbell-Lendrum D, Steenland NK. Occupational noise: assessing the burden of disease from work-related hearing impairment at national and local levels. Geneva: World Health Organization; 2004 [citado 2015 abr 28]. (WHO Environmental Burden of Disease Series, 9). Disponível em: http://www.who.int/quantifying_ehimpacts/publications/en/ebd9.pdf

k Instituto Brasileiro de Geografia e Estatística. Cidades. Brasília (DF); 2014 [citado 2014 jun 18]. Disponível em: http://www.cidades.ibge.gov.br/xtras/home.php

l Ministério do Trabalho e Emprego, Secretaria de Saúde e Segurança no Trabalho. Portaria nº 19 de 9 de abril de 1998. Diretrizes e parâmetros mínimos para avaliação a acompanhamento da audição em trabalhadores expostos a níveis de pressão sonora elevados. *Diario Oficial Uniao*. 22 abr 1998; Seção 1:64-6.
